# Supplementary material for: Feasibility and preliminary efficacy of a virtual reality intervention targeting distress and anxiety in primary brain tumor patients at the time of clinical evaluation: Study protocol for a phase 2 clinical trial
Source: Res Sq. 2023 Feb 16:rs.3.rs-2521990. Preprint. [Version 1] doi: 10.21203/rs.3.rs-2521990/v1 (PMC9980195; doi:10.21203/rs.3.rs-2521990/v1)
Supplement: 1 [file NIHPPrs2521990v1-supplement-1.pdf]

Supplementary Files

This is a list of supplementary files associated with this preprint. Click to download.

- VRmethodspapersupplementssubmission.docx
- Tables12.docx
